# Metabolic engineering of *Acinetobacter baylyi* ADP1 for naringenin production

**DOI:** 10.1016/j.mec.2024.e00249

**Published:** 2024-10-31

**Authors:** Kesi Kurnia, Elena Efimova, Ville Santala, Suvi Santala

**Affiliations:** Faculty of Engineering and Natural Sciences, Tampere University, Hervanta Campus, 33720, Tampere, Finland

**Keywords:** Naringenin, *Acinetobacter baylyi* ADP1, *p-*coumaric acid, Malonyl-CoA

## Abstract

Naringenin, a flavanone and a precursor for a variety of flavonoids, has potential applications in the health and pharmaceutical sectors. The biological production of naringenin using genetically engineered microbes is considered as a promising strategy. The naringenin synthesis pathway involving chalcone synthase (CHS) and chalcone isomerase (CHI) relies on the efficient supply of key substrates, malonyl-CoA and *p*-coumaroyl-CoA. In this research, we utilized a soil bacterium, *Acinetobacter baylyi* ADP1, which exhibits several characteristics that make it a suitable candidate for naringenin biosynthesis; the strain naturally tolerates and can uptake and metabolize *p*-coumaric acid, a primary compound in alkaline-pretreated lignin and a precursor for naringenin production. *A. baylyi* ADP1 also produces intracellular lipids, such as wax esters, thereby being able to provide malonyl-CoA for naringenin biosynthesis. Moreover, the genomic engineering of this strain is notably straightforward. In the course of the construction of a naringenin-producing strain, the *p*-coumarate catabolism was eliminated by a single gene knockout (Δ*hcaA*) and various combinations of plant-derived CHS and CHI were evaluated. The best performance was obtained by a novel combination of genes encoding for a CHS from *Hypericum androsaemum* and a CHI from *Medicago sativa,* that enabled the production of 17.9 mg/L naringenin in batch cultivations from *p*-coumarate. Furthermore, the implementation of a fed-batch system led to a 3.7-fold increase (66.4 mg/L) in naringenin production. These findings underscore the potential of *A. baylyi* ADP1 as a host for naringenin biosynthesis as well as advancement of lignin-based bioproduction.

## Highlights

• *Acinetobacter baylyi* ADP1 was employed as a novel host for naringenin biosynthesis.
• The natural metabolism of aromatic compounds in ADP1 was exploited.
• Lignin-related *p-*coumaric acid provides a suitable precursor for synthesis in ADP1.
• A novel combination of pathway enzymes CHS-CHI resulted in highest naringenin titer.

## 1 Introduction

Flavonoids are an important class of natural products widely found in the plant kingdom (Panche et al., 2016; Pandey et al., 2016). They belong to a class of plant polyphenols, comprising a family of more than 9000 compounds such as flavones, flavanols, isoflavones, anthocyanins, and chalcones (Panche et al., 2016; Zhang et al., 2021; Li et al., 2022). They possess a wide array of valuable applications in the realms of health and pharmaceuticals, encompassing attributes such as anticancer, antioxidant, anti-inflammatory, and antiviral properties, along with recognized neuroprotective and cardioprotective effects (Salehi et al., 2019; Zhang et al., 2021; Cai et al., 2023). Among various classes, one of the important flavonoids is a flavanone naringenin, which occupies a central position as the primary C_15_ intermediate in the flavonoid biosynthesis pathway (Hwang et al., 2021; Zhang et al., 2021). This compound has the carbon framework common to flavonoids (C6−C3−C6) and functions as a precursor to other flavonoids, including flavanones, flavanols, and isoflavonoids (Li et al., 2022; Liu et al., 2022). In addition, a recent in vitro test study has indicated that naringenin could potentially offer a promising strategy for the treatment of COVID-19 (Tutunchi et al., 2020; Clementi et al., 2021).

Traditionally, the production of flavonoid and flavonoid-derived compounds has relied heavily on their isolation from plants, necessitating extensive separation and purification efforts (Koopman et al., 2012; Ye et al., 2023). This approach has proven to be neither cost-effective nor sustainable as a source of flavonoids. Moreover, the conventional method has faced limitations due to the low yield obtained from natural sources (Karim et al., 2018; Hwang et al., 2021). To overcome these challenges, numerous attempts have been undertaken to synthesize naringenin in microbial systems.

Due to the abundance of genetic tools, conventional workhorses such as *Escherichia coli*, *Corynebacterium glutamicum,* and *Saccharomyces cerevisiae*, have been previously employed in the production of flavonoids (Santos et al., 2011; Koopman et al., 2012; Wu et al., 2014, Wu et al., 2022; Milke et al., 2019; Gomes et al., 2024). In microbial hosts, naringenin can be produced from one molecule of *p*-coumaroyl-CoA and three molecules of malonyl-CoA via a naringenin chalcone intermediate (Gao et al., 2020; Liu et al., 2022). For instance, Zhou et al. (2021) produced naringenin from glucose and glycerol in *E. coli* by introducing a chalcone synthase (CHS) from *Petunia hybrida* and a chalcone isomerase (CHI) from *Medicago sativa.* Cells were also supplemented with palmitate and stearate, which were speculated to increase the amount of malonyl-CoA for the synthesis. In another study by Milke et al. (2019), CHS and CHI from *P. hybrida* were expressed in *C. glutamicum* to produce naringenin from glucose. To increase the malonyl-CoA availability, they regulated the fatty acid synthesis and reduced the flux into the tricarboxylic acid cycle (TCA cycle) by metabolic engineering.

Due to the challenges related to the malonyl-CoA availability, the potential of oleaginous microorganisms in the production of flavonoids is being increasingly investigated. These organisms can produce lipids exceeding 20% of their cell dry weight, which implies the potential to overproduce malonyl-CoA. Recently, Zan et al. (2024) engineered a non-model organism *Mucor circinelloides* to produce naringenin by a heterologous pathway. Despite the result that the obtained titer was relatively low (2.2 mg/L), the work nicely demonstrates the potential of leveraging the naturally active anabolic pathways for malonyl-CoA synthesis.

Many of the previously described naringenin production systems rely on the utilization of glucose and/or glycerol as substrates for the synthesis (Santos et al., 2011; Koopman et al., 2012; Hwang et al., 2023). However, the key naringenin precursor, *p-*coumaric acid, represents a major compound of alkaline-pretreated lignin (APL), which could serve as a renewable feedstock for biocatalysts (Vardon et al., 2015; Incha et al., 2020; Weiland et al., 2022). Despite being the second most abundant biopolymer from terrestrial plants, lignin remains significantly underutilized (Becker and Wittmann, 2019; Yu et al., 2023). For instance, chemical pulping operations generate more than 50 million tons of technical lignin as by-products, with most of this being burnt as low-quality fuels (less than $50/dry ton) (Balakshin et al., 2021). Consequently, utilizing lignin-derived aromatics for microbial naringenin production could serve as a sustainable means to utilize natural resources while concurrently enhancing the competitiveness of the lignocellulose-based biorefinery processes.

*Acinetobacter baylyi* ADP1 emerges as a promising candidate for the biological conversion of lignin-derived compounds, due to its capability to uptake and utilize aromatic substances through the *β*-ketoadipate pathway (Harwood and Parales, 1996; Bleichrodt et al., 2010; Salvachúa et al., 2015; Luo et al., 2019; Biggs et al., 2020). The strain can also tolerate and grow on significantly high concentrations of lignin-related aromatics (Luo et al., 2022b). Moreover, we have previously demonstrated *A. baylyi* ADP1 to be a potential host for the overproduction of intracellular storage lipids, namely wax esters (Santala et al., 2021, Santala et al., 2018; Salmela et al., 2019; Luo et al., 2020), also from lignin-related compounds (Salmela et al., 2019; Liu et al., 2024). By overexpressing only the fatty acyl-CoA reductase *acr1,* the lipid production of ADP1 could be significantly increased, resulting in up to 27% wax esters of cell dry weight (Luo et al., 2020). This indicates the potentially high supply of malonyl-CoA in ADP1 for synthesis pathways. Additionally, *A. baylyi* ADP1 is highly engineerable and naturally competent, which facilitates swift and targeted genomic manipulations (Elliott and Neidle, 2011; Biggs et al., 2020; Santala and Santala, 2021) and evolution-based engineering (Tumen-Velasquez et al., 2018; Bedore et al., 2023). Recently, Biggs and Tyo (2023) demonstrated the synthesis of vanillin-glucoside and resveratrol from lignin-related compounds in engineered *A. baylyi* ADP1 (Biggs and Tyo, 2023). Taken together, these features highlight ADP1 as an ideal host for the production of flavonoid-like products from sustainable resources.

**Fig. 1:**
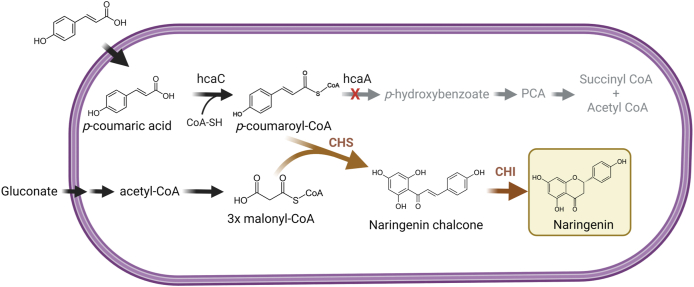
Schematic representation of heterologous production of naringenin in *A. baylyi* ADP1. Black arrows indicate the native pathways. Grey arrows indicate pathway abolished by deletion of a bifunctional hydratase/lyase (*hcaA*), and brown arrows indicate naringenin production pathway. Abbreviations: protocatechuate (PCA), chalcone synthase (CHS), chalcone isomerase (CHI). Created with BioRender.com. (For interpretation of the references to colour in this figure legend, the reader is referred to the Web version of this article.)

In this work, we established naringenin production in *A. baylyi* ADP1 (Fig. 1). We exploited the strain's native ability to utilize *p*-coumaric acid as a substrate and established a non-native pathway for naringenin synthesis. In addition, we implemented a fed-batch process to improve naringenin production, resulting in a notable improvement in naringenin titer. This study demonstrates *A. baylyi* ADP1 as a promising host for biological valorization of lignin-related compounds into high-value flavonoids.

## 2 Materials and methods

### 2.1 Bacterial strains and culture conditions

*Escherichia coli* XL1-Blue (Stratagene, USA) was used for the plasmid constructions and amplifications. Strains were maintained on low-salt (1 g/L NaCl) Lysogeny broth (LB) or LB agar containing antibiotics for plasmid selection. *A. baylyi* ADP1 (DSM 24193, DSMZ, Germany) was used for naringenin production and pathway engineering. *A. baylyi* ADP1 and derived strains were routinely grown aerobically at 30 °C in low-salt LB or Minimal salts medium (MSM). For grown-on plates, 1.5% agar (w/v) was added. Corresponding antibiotics were added to the media when necessary (100 μg/mL ampicillin, 25 μg/mL chloramphenicol, 50 μg/mL kanamycin, and gentamycin 15 μg/mL). All growth and production experiments were performed using MSM (per Liter; 3.88 g K_2_HPO_4_, 1.63 g NaH_2_PO_4_, 2.0 g (NH_4_)_2_SO_4_, pH 7.0). The MSM was supplemented with a trace elements solution (per Liter; 10 mg/L ethylenediaminetetraacetic acid (EDTA), 0.1 g/L MgCl_2_∙6H_2_O 2 mg/L ZnSO_4_∙7H_2_O, 1 mg/L CaCl_2_∙2H_2_O, 5 mg/L FeSO_4_∙7H_2_O, 0.2 mg/L, Na_2_MoO_4_∙2H_2_O, 0.2 mg/L CuSO_4_∙5H_2_O, 0.4 mg/L CoCl_2_∙6H_2_O, 1 mg/L MnCl_2_∙2H_2_O). The stock solution of *p*-coumaric acid was prepared as potassium salt with a concentration of 200 mM (pH 8.2–8.3) as described previously (Luo et al., 2022b). Cerulenin (Sigma Aldrich) was dissolved in ethanol as previously described (Schwanemann et al., 2023). Sodium malonate and naringenin were purchased from Sigma Aldrich.

### 2.2 Genetic modifications

Primers were synthesized by Thermo Scientific and routine PCR amplifications were carried out using Phusion High Fidelity DNA Polymerase (New England Biolabs, UK). Chromosomal modifications were engineered in *A. baylyi* ADP1 by natural transformation of recipient strains with linear DNA fragments as described previously (Luo et al., 2020, 2022a). First, regions of approximately 500 bp-1000 bp upstream (R1) and downstream (R2) of the target genes were amplified from the genomic DNA of *A. baylyi* ADP1. The R1 and R2 were constructed with *tdk-kan*^*R*^ cassette by OE-PCR and transformed to *A. baylyi* ADP1 as described previously (De Berardinis et al., 2008). The rescue cassette (1–2 kb) was introduced to generate a clean knock-out. *A* successful deletion in *A. baylyi* ADP1 was screened on negative selection LB plates with 200 μg/mL 3-azido-2′,3′-dideoxythymidine (AZT) + 0.1% glucose. Genotypes were then confirmed by colony PCR with OneTaq (New England Biolabs, UK) and selected for antibiotic sensitivity. Detailed information regarding all strains and primers used in this study is provided in Table S1 and Table S2.

### 2.3 Pathway construction

Genes encoding malonyl-CoA synthetase from *Rhizobium trifolii* (matB, KF765783.1), chalcone synthase (CHS) from *Petunia X hybrida* (PhCHS, KF765781.1), *Huperzia serrata* (HsPKS1, DQ979827.1), *Hypericum androsaemum* (HaCHS, AF315345.1) and genes encoding chalcone isomerase (CHI) from *Pueraria lobata* (PlCHI, D63577.1) and *Medicago sativa* (MsCHI, KF765782.1) were used in this study. Those genes were synthesized and codon-optimized for ADP1 by GenScript Biotech (Netherlands) with appropriate restriction sites and ribosomal binding sites (BBa_B0034). The naringenin plasmids were constructed as follows: first, each CHS gene was cloned to pBAV1C-chn (Luo et al., 2019) with BioBrick assembly standard using restriction sites XbaI/SpeI and PstI. Subsequently, the resulting plasmid was digested with XbaI and PstI. The CHI gene was cloned downstream of the CHS gene, resulting in the creation of plasmids pNAR1, pNAR2, pNAR3, pNAR4, pNAR5, and pNAR6.

The integration of *matB* from *R. trifolii* into the genome was facilitated by previously described gene integration cassette (Santala et al., 2011a; Lehtinen et al., 2018). The *matB* gene was cloned to integration cassette by utilizing NdeI/XhoI restriction sites. This construct contains two antibiotic markers, *Cm*^*R*^ and *Kan*^*R*^, allowing for selection in *E. coli* and *A. baylyi* ADP1, respectively. Furthermore, this cassette enables the replacement of the genes pyruvate dehydrogenase (*poxB*), homocysteine synthase (*metY*) and fatty acyl-CoA reductase (*acr1*) (ACIAD3383-3381), with the *matB* gene. Notably, the deletion of the *acr1* gene eliminates ADP1's ability to produce wax esters, whereas *poxB* and *metY* have been previously recognized to be neutral deletion sites in terms of the growth of ADP1 (Santala et al., 2011b; Luo et al., 2020). The constructed plasmids were transformed into *E. coli* XL-1 cells and transformants were selected on LB plates with 25 μg/mL chloramphenicol. Verified plasmids were then transformed into *A. baylyi* ADP1 using natural transformation. All plasmid inserts and integration were verified by sequencing (Macrogen, the Netherlands). The gene sequences can be found in Supplementary Table S4.

### 2.4 Small-scale naringenin production and fed-batch fermentation

To monitor naringenin production, single colonies were inoculated into 14 mL tubes with 5 mL MSM containing 50 mM gluconate and 0.2% casamino acids. The precultures were incubated at 30 °C at 300 rpm. After overnight cultivation, the precultures were diluted and transferred to a 100 mL flask containing 20 mL MSM with 50 mM gluconate and 0.2% casamino acids at an initial OD_600_ of 0.7–0.8. Potassium salt of *p*-coumaric acid was added at a concentration 2.5 mM, and cyclohexanone at 5 μM was used to induce heterologous pathway expression. All cultivations were performed in triplicate and were allowed to proceed for 72 hours. ADP1 containing empty plasmid was used as a control.

The fed-batch culture was performed in a 250 mL mini bioreactor (Applikon Biotechnology, The Netherlands) with a 50 mL initial volume. Prior to inoculation, the process parameters were set 200 rpm stirrer speed, aeration, and temperature 30 °C. A fresh colony of the constructed derivatives was grown into MSM containing 50 mM gluconate and 0.2% casamino acids. Subsequently, the culture was transferred to a bioreactor at an initial OD_600_ 0.7–0.8. The feed solution (50 mM gluconate, 0.2% casamino acids, and 3.2 mM *p*-coumaric acid in MSM) was fed at 3.7 mL per hour for 48 hours. Cyclohexanone was added at a concentration 0.5 μM to ensure consistent gene expression (Fig. S1). The utilization of gluconate and casamino acids by the cells causes an increase in the medium's pH. Therefore, the pH of the fermentation was maintained at 7.5 through the addition of 1 M H_3_PO_4_. Additionally, if necessary, the 10% antifoam A (Fluka Analytical) was added. Samples (2 mL) were taken in aseptic conditions throughout the duration of the cultivation to measure cell growth, *p*-coumaric acid, and naringenin. The bioreactor experiments were conducted in duplicate.

### 2.5 Analytical methods

Optical density was measured at 600 nm using an Ultrospec 500 Pro (Amersham Biosciences). For measurement of naringenin, the fermentation broth was mixed with an equal volume of ethyl acetate, vortexed for 30 sec, and rotated at room temperature for 2 hours. After centrifugation, the upper layer was collected, and the solvent was allowed to evaporate overnight. The dried samples were then resuspended in methanol for analysis with HPLC. For measurement of *p-*coumaric acid, the culture was centrifuged at 20,000g for 5 min. The supernatant was taken and filtered with syringe filters (CHROMAFIL® PET, PET-45/25, Macherey–Nagel, Germany). HPLC analysis of *p*-coumaric acid and naringenin was performed using Shimadzu LC-40D instrument (Japan) equipped with a photo diode array detector Shimadzu SPD-M40 (Japan). The compounds were analyzed on the column Luna C18(2) 4.6 × 150 mm, 100 Å (Phenomenex, USA) at 40 °C. The mixture 0.1% formic acid: methanol (6/4, v/v) was used as eluent with the flow rate 1 mL/min. The external standard method was used to calculate concentrations of *p*-coumaric acid and naringenin. The chemical standards were purchased from Sigma. Peaks of *p*-coumaric acid and naringenin were detected at 288 nm with retention times 4.29 min and 16.44 min, respectively.

## 3 Result and discussion

### 3.1 *Acinetobacter baylyi* ADP1 as a naringenin production platform

*A. baylyi* ADP1 possesses efficient tolerance and utilization of lignin-related compounds, such as *p*-coumaric acid (Bleichrodt et al., 2010; Harwood and Parales, 1996), which is a precursor for naringenin. In addition, the ability to accumulate intracellular lipids associated with potentially high availability of malonyl-CoA for syntheses pathways (Luo et al., 2020) and the straightforward engineering renders *A. baylyi* ADP1 as an optimal candidate for the production of flavonoids, such as naringenin.

**Fig. 2:**
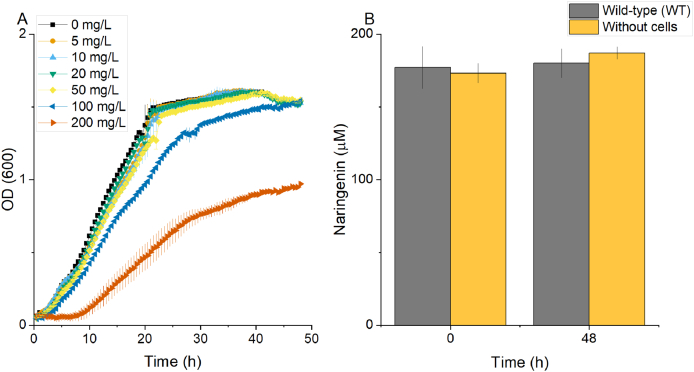
Assessment of *A. baylyi* ADP1 growth in the presence of naringenin. A) Growth of *A. baylyi* ADP1 wild-type (WT) in the presence of an increasing concentration of naringenin in MSM with 50 mM gluconate and 0.2% casamino acids. B) Stability of naringenin in *A. baylyi* ADP1 WT culture. Cells were incubated in MSM with 200 μM (54.5 mg/L) naringenin. Error bars represent the mean ± s.d of three biological replicates.

Since naringenin has been reported to have antimicrobial effects (Lather et al., 2020; Cai et al., 2023), we initially assessed the tolerance of *A. baylyi* ADP1 towards naringenin by culturing the strain in a mineral salts medium (MSM) containing 50 mM gluconate and 0.2% casamino acids with varying concentrations of naringenin (Fig. 2A). The cells were able to grow in the presence of all studied naringenin concentrations; growth was not affected by up to 50 mg/L of naringenin, while 200 mg/L of naringenin resulted in extended lag phase in ADP1. This concentration is relatively low considering the highest naringenin levels that have been previously produced by microbes. For example, Mao et al. (2023) produced 2.05 g/L of naringenin without observed growth defect in engineered *S. cerevisiae* (Mao et al., 2023). However, the naringenin tolerance can be potentially improved by for example adaptive laboratory evolution strategies, as previously successfully demonstrated for ADP1 (Luo et al., 2022b). We also confirmed that *A. baylyi* ADP1 is unable to degrade naringenin (Fig. 2B); no obvious decrease in naringenin concentration was observed after 48 hours fermentation. The above results indicated that *A. baylyi* ADP1 can potentially be harnessed for naringenin production.

### 3.2 Screening an optimal naringenin production module

Naringenin is the starting point for many flavonoid functionalization chemistries (Hwang et al., 2021; Zhang et al., 2021). It is synthesized via the phenylpropanoid metabolic pathway in plants, requiring one molecule of *p*-coumaroyl-CoA and three molecules of malonyl-CoA, catalyzed by the enzyme chalcone synthase. Our aim was to construct the naringenin production pathway from *p*-coumaric acid via chalcone intermediate. First, given that *A. baylyi* ADP1 naturally generates *p*-coumaroyl-CoA as part of its *p-*coumaric acid catabolism, we performed a genetic knockout of the downstream gene in the native catabolic pathway. Specifically, we deleted the *hcaA* gene, which encodes a bifunctional hydratase/lyase responsible for converting the thioester derivative to an aldehyde intermediate (Parke and Ornston, 2004). The rationale behind the deletion of the *hcaA* gene is to increase the availability of *p*-coumaroyl-CoA, a crucial precursor for naringenin production and thereby increasing the product yield. By knocking out *hcaA*, we aimed to prevent *A. baylyi* ADP1 from diverting these precursors towards biomass production. Subsequent cultivation experiments confirmed that the *A. baylyi* ADP1 Δ*hcaA,* designated as ASA800 was incapable of utilizing *p-*coumaric acid as the sole carbon source (Fig. S2).

**Fig. 3:**
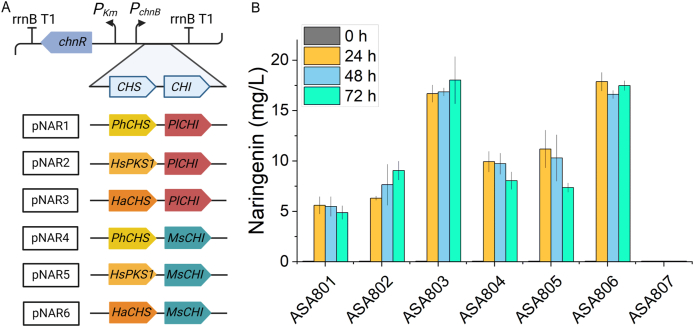
Naringenin production modules in constructed strains. A) Plasmid construction for CHS and CHI expression from different plant species. B) Naringenin production by ASA801-ASA807 in flasks supplemented with 2.5 mM *p-*coumaric acid (potassium salt). pBAV1C-chn was used as an empty plasmid. The expression was regulated by inducible promoter P_*ChnB*_. Error bars represent the mean ± s.d of three biological replicates. Created with BioRender.com.

Subsequently, we proceeded to establish a synthetic pathway and confirmed the viability of employing ASA800 as the host organism for flavonoid production. To construct the naringenin production module, two genes, chalcone synthase (CHS) and chalcone isomerase (CHI) are required. In this study, we introduced CHS originating from various plants, *Petunia X hybrida* (PhCHS)*, Huperzia serrata* (HsPKS1), and *Hypericum androsaemum* (HaCHS), that accept *p*-coumaroyl-CoA as a substrate (Liu et al., 2003; Wu et al., 2014; Palmer et al., 2020). In addition, CHI which is responsible for the further isomerization of chalcone to flavanone, was selected from *Pueraria* *lobata* (PlCHI) and *Medicago sativa* (MsCHI). The CHS-CHI genes were previously expressed for naringenin or other flavonoids production in *E. coli* (Wu et al., 2014; Van Brempt et al., 2022), *Saccharomyces cerevisiae* (Lyu et al., 2019; Mejía-Manzano et al., 2024), *Yarrowia lipolytica* (Palmer et al., 2020) and *Corynebacterium glutamicum* (Kallscheuer et al., 2016). Here the assembly of these orthologues resulted in the generation of six recombinant pathways in which HaCHS – MsCHI represents a novel combination (Fig. 3A). To ensure robust expression of the heterologous genes, genes were expressed in the plasmid pBAV1C-chn under cyclohexanone-inducible promoter *ChnR*/P_ChnB_ (Luo et al., 2019). The constructed plasmids (pNAR1-pNAR6) were then transformed to ASA800, resulting in six naringenin-producing strains, designated as ASA801-ASA806, respectively. The ADP1 Δ*hcaA* harboring empty plasmid pBAV1C-chn (ASA807) was used as a control. The ASA801-ASA807 were then cultivated in MSM with 50 mM gluconate, 0.2% casamino acids, and 2.5 mM *p-*coumaric acid for 72 hours; gluconate was provided for the cells to support cell growth and to provide acetyl-CoA for malonyl-CoA synthesis. All tested combinations were found to be functional and produce naringenin. However, the different combinations of naringenin production modules resulted in varying naringenin titers ranging from 5.6 mg/L to 17.9 mg/L (Fig. 3B). The highest titers were reached already after 24 hours cultivations by cells harboring pNAR3 (ASA803) and pNAR6 (ASA806), yielding 16.7 mg/L and 17.9 mg/L, respectively. Interestingly, both these production strains contained HaCHS, indicating that CHS mainly dictates the efficiency of the naringenin production module in the studied combinations. Thus, these results demonstrated the feasibility of using *A. baylyi* ADP1 as the chassis for de novo synthesis of naringenin.

### 3.3 Toxicity effect caused by *p-*coumaric acid in ADP1 naringenin producing strain

**Fig. 4:**
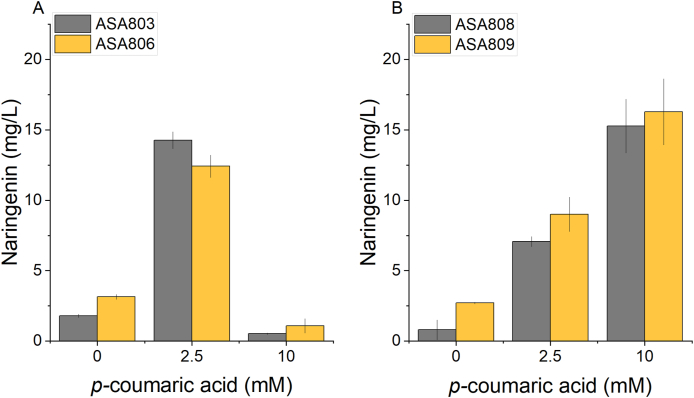
Effect of *p-*coumaric acid concentration on naringenin production in A) ASA803 and ASA806, B) ASA808 and ASA809. Cells were cultivated in MSM with 50 mM gluconate, 0.2% casamino acids, and different concentrations of *p*-coumaric acid (potassium salt). Gene expressions were induced with 5 μM cyclohexanone. Samples for analysis were taken after 24 h. Error bars represent the mean ± s.d of three biological replicates.

In a previous research (Parke and Ornston, 2004), it was highlighted that the deletion of the hydratase/lyase gene *hcaA* abolishes the ability of *Acinetobacter* cells to grow on the three hydroxycinnamates (caffeate, *p*-coumarate, and ferulate). Furthermore, this genetic alteration also inhibits cell growth in the presence of these compounds even in concentrations as low as 100 μM (caffeate), 10 μM (*p-*coumarate) and 1 mM (ferulate) (Parke and Ornston, 2003, 2004). The deletion of *hcaA* causes the buildup of hydroxycinnamoyl-CoA in the presence of hydroxycinnamates (Parke and Ornston, 2004; Biggs and Tyo, 2023). Introducing mutation to *hcaC*, a hydroxycinnamate:coenzyme A (CoA) SH ligase, rescued the Δ*hcaA* cells to grow in the presence of hydroxycinnamates (Parke and Ornston, 2004), indicating that the hydroxycinnamoyl-CoA is more toxic than its hydroxycinnamate counterpart. Similar toxic effects by *p-*coumaroyl-CoA have also been observed in *Pseudomonas putida* KT2440 (Incha et al., 2020) and *Saccharomyces cerevisiae* (Liu et al., 2022). Thus, we investigated the impact of *p-*coumaric acid concentration on our naringenin production strains ASA803 and ASA806. The strains were tested with two different *p*-coumaric acid concentrations (2.5 mM and 10 mM of *p*-coumaric acid) and naringenin levels were assessed after 24 hours of cultivation. We observed that *p-*coumaric acid concentration of 10 mM resulted in decreased naringenin production in both ASA803 and ASA806, indicating a toxic effect of this compound (Fig. 4A). This observation supports the notion that a high concentration of the *p-*coumaroyl-CoA intermediate is toxic for ASA800.

To further investigate the potentially negative effect of the *hcaA* deletion, we conducted a test with the wild-type (WT) ADP1 carrying pNAR3 (ASA808) and pNAR6 (ASA809) for comparison (Fig. 4B). We hypothesized that a potential ‘pulling effect’ generated by native *p*-coumarate utilization pathway might have a positive effect on the naringenin accumulation. Indeed, we observed that at a high *p-*coumaric acid concentration (10 mM), a higher naringenin titer was obtained compared to the Δ*hcaA* strains. However, at a lower *p-*coumaric acid concentration (2.5 mM), the titer was lower in the WT strains, indicating that a significant amount of the *p-*coumaric acid is channeled to the catabolic pathway rather than the naringenin synthesis pathway. Although the deletion of *hcaA* increases sensitivity to *p-*coumaric acid, the results indicate that the deletion leads to a higher availability of *p*-coumaroyl-CoA, a precursor for naringenin, and therefore potentially higher yield. Thus, for further experiments, we chose to continue with ASA803 and ASA806 using lower concentrations of *p-*coumaric acid (2.5 mM), for which the tolerance was found to be sufficient.

### 3.4 Effect of deletion of malonate utilization pathway and addition of FAS cerulenin on naringenin production

Malonyl-CoA constitutes a vital precursor for the biosynthesis of flavonoids (Wu et al., 2015; Milke et al., 2019; Peng et al., 2023). It serves as a co-substrate for CHS, and it plays crucial role in naringenin production (Wu et al., 2022; Xu et al., 2011). Therefore, the availability of malonyl-CoA can be the principal limiting factor in the flavonoid synthesis. Several efforts have been made to increase the pool of this important precursor molecule. For example, Wu et al. (2022) introduced *matB* and *matC* genes from *Rhizobium trifolii* into *Corynebacterium glutamicum*, resulting in a remarkable 35-fold increase in naringenin production. In this context, we investigated two strategies for increasing the supply of malonyl-CoA to improve naringenin yields. In the first approach, we obstructed the malonate degradation pathway in ADP1 and introduced *matB* gene from *Rhizobium trifolii*, the enzyme responsible for the conversion of malonate to malonyl-CoA. In *A. baylyi* ADP1, a cluster of *mdc* genes facilitates the utilization of malonate as a carbon source (Stoudenmire et al., 2017). Thus, in order to prevent further malonate degradation, the malonate pathway in ADP1 was eliminated, involving the deletion of *mdcA, mdcB, mdcC, mdcD, mdcE, mdcG*, and *mdcH*. The malonate transporter (*mdcLM*), however, was not deleted, allowing malonate to be imported into the cell. We then incorporated codon-optimized *matB* from *R. trifolii* into the genome using a previously described gene integration cassette (Lehtinen et al., 2018). This cassette also removes the strain's ability to produce wax ester due to the deletion of the gene encoding for the fatty acyl-CoA reductase *acr1* (ACIAD3383) (Santala et al., 2014), thus preventing the direction of malonyl-CoA towards wax ester synthesis. Subsequently, we conducted experiment to assess naringenin production in MSM supplemented with 50 mM gluconate, 0.2% casamino acid, 2.5 mM *p-*coumaric acid and 15 mM sodium malonate. Regrettably, the Δ*hcaA* Δ*mdcABCDEGH* Δ*poxB* Δ*metY* Δ*acr1::matB* harboring pNAR3 (ASA810) failed to grow on this medium at the studied conditions. A clear growth defect was also observed in Δ*mdcABCDEGH* Δ*poxB* Δ*metY* Δ*acr1::matB* pNAR3 (ASA811) compared to ASA808 (Fig. S3). Thus, the integration of *matB* did not increase overall naringenin production. It appears that the introduced genetic modifications in combination detrimentally affect the growth of ADP1 and would thus require more investigation.

The second approach involved the utilization of cerulenin, a compound known for its inhibitory action on fatty acid biosynthesis. Cerulenin binds to the β-ketoacyl-acyl carrier protein (ACP) synthase of the fatty acid machinery, thereby restricting the depletion of malonyl-CoA for fatty acid biosynthesis (Santos et al., 2011). Supplementation of cerulenin improved resveratrol titers in *C. glutamicum* (Kallscheuer et al., 2016)*,* flaviolin titers in *Pseudomonas* *taiwanensis* (Schwanemann et al., 2023)*,* and naringenin titers in *E. coli* (Santos et al., 2011). Given the identical CHS gene in both pNAR3 and pNAR6 and the high cost of cerulenin, in this study, we conducted our experiment solely with ASA803. As depicted in Fig. S4A, the growth of our strain remained unaffected by a 50 μM concentration of cerulenin, a factor advantageous for both naringenin yield and productivity. In prior research, it was observed that cerulenin supplementation negatively impacted the growth in *C. glutamicum* (Kallscheuer et al., 2016) and *E. coli* (Leonard et al., 2008) while producing higher amount of flavonoids. However, ASA803 exhibited lower naringenin production in the presence of cerulenin (Fig. S4B). This parallels the finding documented by Dunstan et al. (2020), regarding the CHS from *Camelia sinensis* (Tea plant), showing a similar outcome (Dunstan et al., 2020). Similar findings were also reported in *Streptomyces albidoflavus* J1074, where cerulenin failed to increase naringenin production Ye et al. (2023). Ferrer et al. (1999) reported that cerulenin is a potent irreversible inhibitor of CHS through its binding to Cys-164, thereby restricting access to the *p*-coumaroyl-binding pocket (Ferrer et al., 1999). Hence, it is plausible that instead of significantly affecting fatty acid biosynthesis, cerulenin might bind to CHS, impending naringenin biosynthesis in *A. baylyi* ADP1.

### 3.5 Naringenin production in a bioreactor

**Fig. 5:**
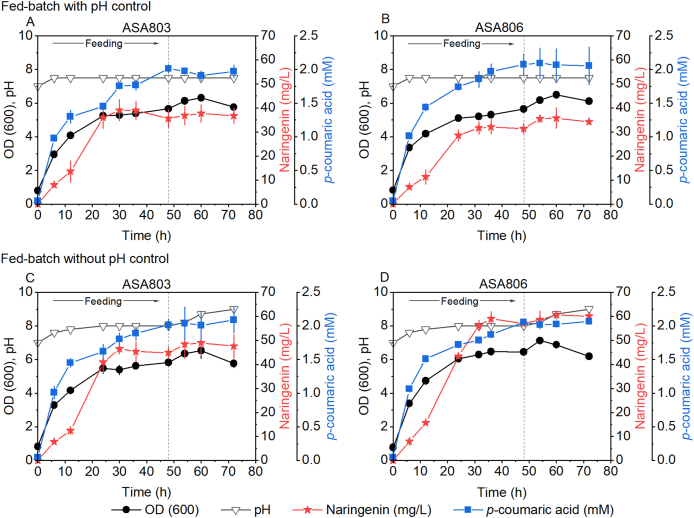
Naringenin production in ASA803 and ASA806 in fed-batch cultivation with pH control (A) and without pH control (B). Cultures initially contained 50 μM of *p-*coumaric acid (potassium salt), 50 mM sodium gluconate, and 0.2% casamino acids. Cultures were fed with a medium containing 50 mM sodium gluconate, 0.2% casamino acids, and 3.2 mM *p*-coumaric acid (potassium salt) for 48 hours (feeding rate 3.7 mL/h). Error bars represent the mean ± s.d of two biological replicates.

Our previous results indicate that the *hcaA* deletion strain is more sensitive to *p*-coumaric acid than the wild-type strain. Therefore, to minimize the impact of high concentration of *p*-coumaric acid, a fed-batch approach was implemented. We studied the performance of the naringenin-producing strains ASA803 and ASA806 in a 250 mL bioreactor set at a pH of 7.5. As a comparative measure, a cultivation in a bioreactor without pH control was performed. Additionally, unlike the flask experiments, a lower inducer concentration (0.5 μM instead of 5 μM of cyclohexanone) was used, as a higher inducer concentration did not increase naringenin production under the studied conditions (Fig. S1). The cyclohexanone-inducible promoter in ADP1 has been previously characterized, with some leakiness observed (Luo et al., 2022a). In this study, the leaky expression of proteins was sufficient to achieve the same level of naringenin production as fully induced cells. However, the expression was more consistent in induced cells compared to uninduced cells, justifying the use of a low inducer concentration. Therefore, cyclohexanone was added at a concentration of 0.5 μM. As shown in Fig. 5A and B, under pH-controlled conditions, the ASA803 and ASA806 cultures produced 36.8 mg/L and 34.2 mg/L of naringenin, respectively. This represented a significant increase in naringenin production compared to the flask experiment, with a 2.2-fold and 1.9-fold enhancement. Without pH control, the pH increased to 8, which resulted in even higher naringenin titers in both strains, namely 47.6 mg/L and 60.0 mg/L for ASA803 and ASA806, respectively (Fig. 5C and D). The improved naringenin titers observed at higher pH levels could be related to potentially reduced toxicity of *p*-coumaric acid due to its deprotonation, which decreases passive transport into cells. Furthermore, the expression of MsCHI from *M. sativa* resulted in an increased naringenin production compared to PhCHI from *P. hybrida*. This finding is supported by the previous results where it was observed that the replacement of PhCHI with MsCHI from *M. sativa* led to an enhanced production of pinocembrin, naringenin, and eriodyctiol (Leonard et al., 2007). To the best of our knowledge, pNAR6 (HaCHS – MsCHI) represents a novel combination as this specific configuration has not been previously expressed in other microbial hosts.

**Fig. 6:**
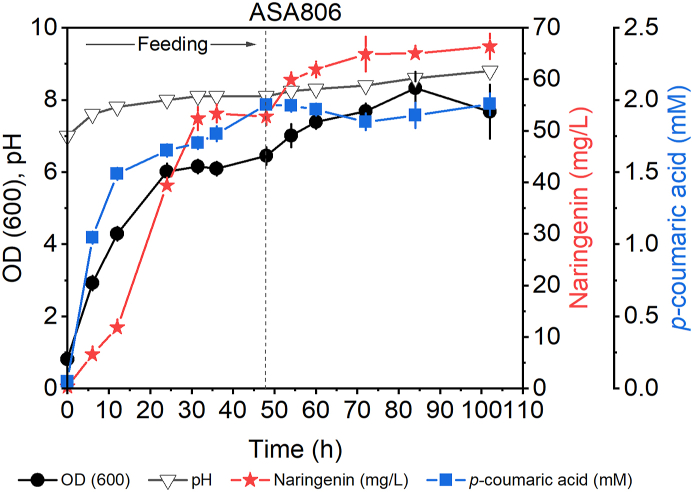
Fed-batch cultivation for naringenin production in higher concentration of gluconate. Cultures initially contained 50 μM of *p*-coumaric acid (potassium salt), 100 mM gluconate, and 0.2% casamino acids. Cultures were fed with a medium containing 100 mM gluconate, 0.2% casamino acids, and 3.2 mM *p-*coumaric acid (potassium salt) for 48 hours (feeding rate 3.7 mL/h). Error bars represent the mean ± s.d of two biological replicates.

Next, we investigated if elevating the gluconate concentration could increase the naringenin production in ASA806. A 100 mM gluconate was used in the feeding medium, and the cultivation was carried out for 102 hours. As shown in Fig. 6, naringenin production was evident already after 6 hours, reaching 52.4 mg/L within 31.5 hours, corresponding to the productivity of 2.2 mg/L/h. The final titer of 66.4 mg/L was attained at the end of the cultivation. Thus, the fed-batch experiments also showed that most of naringenin was produced already during the first 31.5 hours. This indicates that the availability of precursors might not be limiting the production in actively growing cells.

In contrast to the study by Incha et al. (2020) in which higher flaviolin concentration with increased glucose supplementation was reported, our engineered strain did not exhibit significantly higher naringenin production with increased supply of gluconate. This finding together with our results related to the experiments with engineered malonate pathway and cerulenin addition might indicate that the cellular malonyl-CoA content was not potentially limiting the naringenin synthesis in the studied conditions. It is also possible that the higher gluconate availability led to the direction of carbon towards the lipid production pathways of *A. baylyi* ADP1, which competes for acetyl-CoA through fatty acid synthesis (Luo et al., 2020; Arvay et al., 2021). This is further supported by the finding that the naringenin yield of ASA806 supplemented with 100 mM gluconate was 0.32 g_naringenin_/g_*p*-coumaric acid_, which was lower in comparison to a previous experiment with the same strain but with lower gluconate supplementation (0.54 g_naringenin_/g_*p*-coumaric acid_) (Table S3).

Although the production metrics obtained in this study are lower than those achieved in highly optimized systems (Mao et al., 2023), our findings represent the initial steps towards establishing high-value product synthesis in *A. baylyi* ADP1 and highlight the potential of this host for naringenin biosynthesis from lignin-related compounds; for example, while only certain aromatic compounds, such as *p*-coumaric acid, can act as direct precursors for product synthesis, other lignin-derived compounds could serve as sources for biomass and malonyl-CoA, eliminating the need for sugar-based substrates. Using *p*-coumaric acid directly as the substrate also simplifies the production pathway and facilitates the characterization of potential new enzyme activities. Our future work will focus on improving the catalytic efficiency of CHS and CHI as well as bioprocess optimization and product tolerance of *A. baylyi* ADP1, to achieve higher volumetric titers of naringenin and potentially other flavanones and flavonoids.

## 4 Conclusions

In this study, we engineered *A. baylyi* ADP1 for the production of naringenin from *p-*coumaric acid. We first eliminated the native catabolic pathway for *p*-coumarate utilization and then investigated several different combinations of CHS and CHI genes for the production. The highest naringenin titer, 66 mg/L, was obtained with the expression of CHS from *Hypericum androsaemum* and CHI from *Medicago sativa* in a fed-batch cultivation. The outcomes of our study demonstrate the potential of *A. baylyi* ADP1 as a host for flavonoid production. In particular, the high engineerability, the native aromatics catabolic pathways, and the natural ability to provide surplus of malonyl-CoA make *A. baylyi* ADP1 an attractive host for the production. While future research efforts are required to solve remaining issues related to insufficient catalytic efficiencies of CHS and CHI, and further increase of the naringenin production metrics, the results achieved in this study mark a significant step towards the development of lignin-based production of value-added compounds in *A. baylyi* ADP1.

## CRediT authorship contribution statement

**Kesi Kurnia:** Writing – review & editing, Writing – original draft, Visualization, Validation, Methodology, Investigation, Formal analysis, Conceptualization. **Elena Efimova:** Writing – original draft, Methodology. **Ville Santala:** Writing – review & editing, Writing – original draft, Supervision, Methodology, Conceptualization. **Suvi Santala:** Writing – review & editing, Writing – original draft, Supervision, Methodology, Funding acquisition, Conceptualization.

## Funding

SS would like to thank the Novo Nordisk Foundation (grant NNF21OC0067758) and the Research Council of Finland (grant no. 347204 and 353587). VS would like to thank the Novo Nordisk Foundation (grant NNF21OC0079579).

## Declaration of competing interest

The authors declare that they have no known competing financial interests or personal relationships that could have appeared to influence the work reported in this paper.

## Data Availability

Data will be made available on request.

## References

[bib1] Arvay E., Biggs B.W., Guerrero L., Jiang V., Tyo K. (2021). Engineering *Acinetobacter baylyi* ADP1 for mevalonate production from lignin-derived aromatic compounds. Metab. Eng. Commun..

[bib2] Balakshin M.Yu, Capanema E.A., Sulaeva I., Schlee P., Huang Z., Feng M., Borghei M., Rojas O.J., Potthast A., Rosenau T. (2021). New opportunities in the valorization of technical lignins. ChemSusChem.

[bib3] Becker J., Wittmann C. (2019). A field of dreams: lignin valorization into chemicals, materials, fuels, and health-care products. Biotechnol. Adv..

[bib4] Bedore S.R., Neidle E.L., Pardo I., Luo J., Baugh A.C., Duscent-Maitland C.V., Tumen-Velasquez M.P., Santala V., Santala S. (2023). Methods in Microbiology.

[bib5] Biggs B.W., Bedore S.R., Arvay E., Huang S., Subramanian H., McIntyre E.A., Duscent-Maitland C.V., Neidle E.L., Tyo K.E.J. (2020). Development of a genetic toolset for the highly engineerable and metabolically versatile *Acinetobacter baylyi* ADP1. Nucleic Acids Res..

[bib6] Biggs B.W., Tyo K.E.J. (2023). Aromatic natural products synthesis from aromatic lignin monomers using *Acinetobacter baylyi* ADP1. bioRxiv.

[bib7] Bleichrodt F.S., Fischer R., Gerischer U.C. (2010). The β-ketoadipate pathway of *Acinetobacter baylyi* undergoes carbon catabolite repression, cross-regulation and vertical regulation, and is affected by Crc. Microbiology.

[bib8] Cai J., Wen H., Zhou H., Zhang D., Lan D., Liu S., Li C., Dai X., Song T., Wang X., He Y., He Z., Tan J., Zhang J. (2023). Naringenin: a flavanone with anti-inflammatory and anti-infective properties. Biomed. Pharmacother..

[bib9] Clementi N., Scagnolari C., D'Amore A., Palombi F., Criscuolo E., Frasca F., Pierangeli A., Mancini N., Antonelli G., Clementi M., Carpaneto A., Filippini A. (2021). Naringenin is a powerful inhibitor of SARS-CoV-2 infection in vitro. Pharmacol. Res..

[bib10] De Berardinis V., Vallenet D., Castelli V., Besnard M., Pinet A., Cruaud C., Samair S., Lechaplais C., Gyapay G., Richez C., Durot M., Kreimeyer A., Le Fèvre F., Schächter V., Pezo V., Döring V., Scarpelli C., Médigue C., Cohen G.N., Marlière P., Salanoubat M., Weissenbach J. (2008). A complete collection of single‐gene deletion mutants of *Acinetobacter baylyi* ADP1. Mol. Syst. Biol..

[bib11] Dunstan M.S., Robinson C.J., Jervis A.J., Yan C., Carbonell P., Hollywood K.A., Currin A., Swainston N., Feuvre R.L., Micklefield J., Faulon J.-L., Breitling R., Turner N., Takano E., Scrutton N.S. (2020). Engineering *Escherichia coli* towards de novo production of gatekeeper (2S)-flavanones: naringenin, pinocembrin, eriodictyol and homoeriodictyol. Synth. Biol..

[bib12] Elliott K.T., Neidle E.L. (2011). *Acinetobacter baylyi* ADP1: transforming the choice of model organism. IUBMB Life.

[bib13] Ferrer J.-L., Jez J.M., Bowman M.E., Dixon R.A., Noel J.P. (1999). Structure of chalcone synthase and the molecular basis of plant polyketide biosynthesis. Nat. Struct. Biol..

[bib14] Gao S., Lyu Y., Zeng W., Du G., Zhou J., Chen J. (2020). Efficient biosynthesis of (2 *S*)-Naringenin from *p* -coumaric acid in *Saccharomyces cerevisiae*. J. Agric. Food Chem..

[bib15] Gomes D., Rodrigues J.L., Rodrigues L.R. (2024). Step-by-step optimization of a heterologous pathway for de novo naringenin production in *Escherichia coli*. Appl. Microbiol. Biotechnol..

[bib16] Harwood C.S., Parales R.E. (1996). The β-KETOADIPATE pathway and the biology of self-identity. Annu. Rev. Microbiol..

[bib17] Hwang H.G., Milito A., Yang J.-S., Jang S., Jung G.Y. (2023). Riboswitch-guided chalcone synthase engineering and metabolic flux optimization for enhanced production of flavonoids. Metab. Eng..

[bib18] Hwang H.G., Noh M.H., Koffas M.A.G., Jang S., Jung G.Y. (2021). Multi-level rebalancing of the naringenin pathway using riboswitch-guided high-throughput screening. Metab. Eng..

[bib19] Incha M.R., Thompson M.G., Blake-Hedges J.M., Liu Y., Pearson A.N., Schmidt M., Gin J.W., Petzold C.J., Deutschbauer A.M., Keasling J.D. (2020). Leveraging host metabolism for bisdemethoxycurcumin production in *Pseudomonas putida*. Metab. Eng. Commun..

[bib20] Kallscheuer N., Vogt M., Stenzel A., Gätgens J., Bott M., Marienhagen J. (2016). Construction of a *Corynebacterium glutamicum* platform strain for the production of stilbenes and (2S)-flavanones. Metab. Eng..

[bib21] Karim N., Jia Z., Zheng X., Cui S., Chen W. (2018). A recent review of citrus flavanone naringenin on metabolic diseases and its potential sources for high yield-production. Trends Food Sci. Technol..

[bib22] Koopman F., Beekwilder J., Crimi B., Van Houwelingen A., Hall R.D., Bosch D., Van Maris A.J., Pronk J.T., Daran J.-M. (2012). De novo production of the flavonoid naringenin in engineered *Saccharomyces cerevisiae*. Microb. Cell Factories.

[bib23] Lather A., Sharma S., Khatkar A. (2020). Naringenin derivatives as glucosamine-6-phosphate synthase inhibitors: synthesis, antioxidants, antimicrobial, preservative efficacy, molecular docking and in silico ADMET analysis. BMC Chem..

[bib24] Lehtinen T., Efimova E., Santala S., Santala V. (2018). Improved fatty aldehyde and wax ester production by overexpression of fatty acyl-CoA reductases. Microb. Cell Factories.

[bib25] Leonard E., Lim K.-H., Saw P.-N., Koffas M.A.G. (2007). Engineering central metabolic pathways for high-level flavonoid production in *Escherichia coli*. Appl. Environ. Microbiol..

[bib26] Leonard E., Yan Y., Fowler Z.L., Li Z., Lim C.-G., Lim K.-H., Koffas M.A.G. (2008). Strain improvement of recombinant *Escherichia coli* for efficient production of plant flavonoids. Mol. Pharm..

[bib27] Li H., Lyv Y., Zhou S., Yu S., Zhou J. (2022). Microbial cell factories for the production of flavonoids–barriers and opportunities. Bioresour. Technol..

[bib28] Liu B., Falkenstein‐Paul H., Schmidt W., Beerhues L. (2003). Benzophenone synthase and chalcone synthase from *Hypericum androsaemum* cell cultures: cDNA cloning, functional expression, and site‐directed mutagenesis of two polyketide synthases. Plant J..

[bib29] Liu C., Choi B., Efimova E., Nygård Y., Santala S. (2024). Enhanced upgrading of lignocellulosic substrates by coculture of *Saccharomyces cerevisiae* and *Acinetobacter baylyi* ADP1. Biotechnol. Biofuels.

[bib30] Liu D., Sica M.S., Mao J., Chao L.F.-I., Siewers V. (2022). A *p* -Coumaroyl-CoA biosensor for dynamic regulation of naringenin biosynthesis in *Saccharomyces cerevisiae*. ACS Synth. Biol..

[bib31] Luo J., Efimova E., Losoi P., Santala V., Santala S. (2020). Wax ester production in nitrogen-rich conditions by metabolically engineered *Acinetobacter baylyi* ADP1. Metab. Eng. Commun..

[bib32] Luo J., Efimova E., Volke D.C., Santala V., Santala S. (2022). Engineering cell morphology by CRISPR interference in *Acinetobacter baylyi* ADP1. Microb. Biotechnol..

[bib33] Luo J., Lehtinen T., Efimova E., Santala V., Santala S. (2019). Synthetic metabolic pathway for the production of 1-alkenes from lignin-derived molecules. Microb. Cell Factories.

[bib34] Luo J., McIntyre E.A., Bedore S.R., Santala V., Neidle E.L., Santala S. (2022). Characterization of highly ferulate-tolerant *Acinetobacter baylyi* ADP1 isolates by a rapid reverse engineering method. Appl. Environ. Microbiol..

[bib35] Lyu X., Zhao G., Ng K.R., Mark R., Chen W.N. (2019). Metabolic engineering of *Saccharomyces cerevisiae* for de novo production of kaempferol. J. Agric. Food Chem..

[bib36] Mao J., Mohedano M.T., Fu J., Li X., Liu Q., Nielsen J., Siewers V., Chen Y. (2023). Fine-tuning of p-coumaric acid synthesis to increase (2S)-naringenin production in yeast. Metab. Eng..

[bib37] Mejía-Manzano L.A., Ortiz-Alcaráz C.I., Parra Daza L.E., Suarez Medina L., Vargas-Cortez T., Fernández-Niño M., González Barrios A.F., González-Valdez J. (2024). *Saccharomyces cerevisiae* biofactory to produce naringenin using a systems biology approach and a bicistronic vector expression strategy in flavonoid production. Microbiol. Spectr..

[bib38] Milke L., Ferreira P., Kallscheuer N., Braga A., Vogt M., Kappelmann J., Oliveira J., Silva A.R., Rocha I., Bott M., Noack S., Faria N., Marienhagen J. (2019). Modulation of the central carbon metabolism of *Corynebacterium glutamicum* improves malonyl‐CoA availability and increases plant polyphenol synthesis. Biotechnol. Bioeng..

[bib39] Palmer C.M., Miller K.K., Nguyen A., Alper H.S. (2020). Engineering 4-coumaroyl-CoA derived polyketide production in *Yarrowia lipolytica* through a β-oxidation mediated strategy. Metab. Eng..

[bib40] Panche A.N., Diwan A.D., Chandra S.R. (2016). Flavonoids: an overview. J. Nutr. Sci..

[bib41] Pandey R.P., Parajuli P., Koffas M.A.G., Sohng J.K. (2016). Microbial production of natural and non-natural flavonoids: pathway engineering, directed evolution and systems/synthetic biology. Biotechnol. Adv..

[bib42] Parke D., Ornston L.N. (2004). Toxicity caused by hydroxycinnamoyl-coenzyme A thioester accumulation in mutants of *acinetobacter* sp. strain ADP1. Appl. Environ. Microbiol..

[bib43] Parke D., Ornston L.N. (2003). Hydroxycinnamate (*hca*) catabolic genes from *acinetobacter* sp. strain ADP1 are repressed by HcaR and are induced by hydroxycinnamoyl-coenzyme A thioesters. Appl. Environ. Microbiol..

[bib44] Peng B., Dai L., Iacovelli R., Driessen A.J.M., Haslinger K. (2023). Heterologous naringenin production in the filamentous fungus *Penicillium rubens*. J. Agric. Food Chem..

[bib45] Salehi B., Fokou P., Sharifi-Rad M., Zucca P., Pezzani R., Martins N., Sharifi-Rad J. (2019). The therapeutic potential of naringenin: a review of clinical trials. Pharmaceuticals.

[bib46] Salmela M., Lehtinen T., Efimova E., Santala S., Santala V. (2019). Alkane and wax ester production from lignin‐related aromatic compounds. Biotechnol. Bioeng..

[bib47] Salvachúa D., Karp E.M., Nimlos C.T., Vardon D.R., Beckham G.T. (2015). Towards lignin consolidated bioprocessing: simultaneous lignin depolymerization and product generation by bacteria. Green Chem..

[bib48] Santala S., Efimova E., Karp M., Santala V. (2011). Real-Time monitoring of intracellular wax ester metabolism. Microb. Cell Factories.

[bib49] Santala S., Efimova E., Kivinen V., Larjo A., Aho T., Karp M., Santala V. (2011). Improved triacylglycerol production in *Acinetobacter baylyi* ADP1 by metabolic engineering. Microb. Cell Factories.

[bib50] Santala S., Efimova E., Koskinen P., Karp M.T., Santala V. (2014). Rewiring the wax ester production pathway of *Acinetobacter baylyi* ADP1. ACS Synth. Biol..

[bib51] Santala S., Efimova E., Santala V. (2018). Dynamic decoupling of biomass and wax ester biosynthesis in *Acinetobacter baylyi* by an autonomously regulated switch. Metab. Eng. Commun..

[bib52] Santala S., Santala V. (2021). *Acinetobacter baylyi* ADP1—naturally competent for synthetic biology. Essays Biochem..

[bib53] Santala S., Santala V., Liu N., Stephanopoulos G. (2021). Partitioning metabolism between growth and product synthesis for coordinated production of wax esters in *Acinetobacter baylyi* ADP1. Biotechnol. Bioeng..

[bib54] Santos C.N.S., Koffas M., Stephanopoulos G. (2011). Optimization of a heterologous pathway for the production of flavonoids from glucose. Metab. Eng..

[bib55] Schwanemann T., Otto M., Wynands B., Marienhagen J., Wierckx N. (2023). A *Pseudomonas taiwanensis* malonyl-CoA platform strain for polyketide synthesis. Metab. Eng..

[bib70] Stoudenmire J.L., Schmidt A.L., Tumen-Velasquez M.P., Elliott K.T., Laniohan N.S., Walker Whitley S., Galloway N.R., Nune M., West M., Momany C., Neidle E.L., Karls A.C. (2017). Malonate degradation in Acinetobacter baylyi ADP1: operon organization and regulation by MdcR. Microbiology.

[bib56] Tumen-Velasquez M., Johnson C.W., Ahmed A., Dominick G., Fulk E.M., Khanna P., Lee S.A., Schmidt A.L., Linger J.G., Eiteman M.A., Beckham G.T., Neidle E.L. (2018). Accelerating pathway evolution by increasing the gene dosage of chromosomal segments. Proc. Natl. Acad. Sci. U.S.A..

[bib57] Tutunchi H., Naeini F., Ostadrahimi A., Hosseinzadeh‐Attar M.J. (2020). Naringenin, a flavanone with antiviral and anti‐inflammatory effects: a promising treatment strategy against COVID ‐19. Phytother Res..

[bib58] Van Brempt M., Peeters A.I., Duchi D., De Wannemaeker L., Maertens J., De Paepe B., De Mey M. (2022). Biosensor-driven, model-based optimization of the orthogonally expressed naringenin biosynthesis pathway. Microb. Cell Factories.

[bib59] Vardon D.R., Franden M.A., Johnson C.W., Karp E.M., Guarnieri M.T., Linger J.G., Salm M.J., Strathmann T.J., Beckham G.T. (2015). Adipic acid production from lignin. Energy Environ. Sci..

[bib60] Weiland F., Kohlstedt M., Wittmann C. (2022). Guiding stars to the field of dreams: metabolically engineered pathways and microbial platforms for a sustainable lignin-based industry. Metab. Eng..

[bib61] Wu J., Du G., Chen J., Zhou J. (2015). Enhancing flavonoid production by systematically tuning the central metabolic pathways based on a CRISPR interference system in *Escherichia coli*. Sci. Rep..

[bib62] Wu J., Zhou T., Du G., Zhou J., Chen J. (2014). Modular optimization of heterologous pathways for de novo synthesis of (2S)-Naringenin in *Escherichia coli*. PLoS One.

[bib63] Wu X., Liu J., Liu D., Yuwen M., Koffas M.A.G., Zha J. (2022). Biosynthesis of eriodictyol from tyrosine by *Corynebacterium glutamicum*. Microb. Cell Factories.

[bib64] Xu P., Ranganathan S., Fowler Z.L., Maranas C.D., Koffas M.A.G. (2011). Genome-scale metabolic network modeling results in minimal interventions that cooperatively force carbon flux towards malonyl-CoA. Metab. Eng..

[bib65] Ye S., Magadán-Corpas P., Pérez-Valero Á., Villar C.J., Lombó F. (2023). Metabolic engineering strategies for naringenin production enhancement in *Streptomyces albidoflavus* J1074. Microb. Cell Factories.

[bib66] Yu X., Yang B., Zhu W., Deng T., Pu Y., Ragauskas A., Wang H. (2023). Towards functionalized lignin and its derivatives for high-value material applications. Ind. Crop. Prod..

[bib67] Zan X., Xu Y., Chen G., Wang L., Yang J., Wen Y., Sun L., Cui F., Sun W., Song Y., Koffas M.A.G. (2024). Biosynthesis of naringenin from *p* -coumaric acid in engineering oleaginous filamentous fungus *Mucor circinelloides*. ACS Food Sci. Technol..

[bib68] Zhang Q., Yu S., Lyu Y., Zeng W., Zhou J. (2021). Systematically engineered fatty acid catabolite pathway for the production of (2 *S*)-Naringenin in *Saccharomyces cerevisiae*. ACS Synth. Biol..

[bib69] Zhou S., Yuan S.-F., Nair P.H., Alper H.S., Deng Y., Zhou J. (2021). Development of a growth coupled and multi-layered dynamic regulation network balancing malonyl-CoA node to enhance (2S)-naringenin biosynthesis in *Escherichia coli*. Metab. Eng..

