# Supplementary material for: Metabolic engineering of Acinetobacter baylyi ADP1 for naringenin production
Source: Metab Eng Commun. 2024 Oct 31;19:e00249. doi: 10.1016/j.mec.2024.e00249 (PMC11568779; doi:10.1016/j.mec.2024.e00249)
Supplement: Multimedia component 1 [file mmc1.docx]

## Supplementary Information

## Metabolic engineering of *Acinetobacter baylyi* ADP1 for naringenin production

Kesi Kurnia^a^, Elena Efimova^a^, Ville Santala^a^, Suvi Santala^a^

^a^Faculty of Engineering and Natural Sciences, Tampere University, Hervanta Campus, 33720 Tampere, Finland





**Fig. S1.** Effect of cyclohexanone for naringenin production in ASA803. Cells were cultured in MSM with 50 mM gluconate, 0.2 % casamino acids, 2.5 mM *p-*coumaric acid (potassium salt) and different concentration of cyclohexanone. Error bars represent the mean ± s.d of three biological replicates.





**Fig. S2.** Growth of *A. baylyi* ADP1 WT (A) and ASA800 (B) in MSM with *p-*coumaric acid (potassium salt) as sole carbon source (0, 2.5, 5, and 10 mM). Error bars represent the mean ± s.d of three biological replicates.





**Fig. S3.** Effect of malonate utilization deletion and Rt*matB* integration on growth (A) and naringenin production (B) in ASA808 and ASA811. Cells were cultured in MSM with 50 mM gluconate, 0.2% casamino acids, 2.5 mM *p-*coumaric acid (potassium salt), and with or without 15 mM sodium malonate. Error bars represent the mean ± s.d of three biological replicates.





**Fig. S4.** Effect of cerulenin on bacterial growth (A) and naringenin titers (B) in ASA803. Cells were cultured in MSM with 50 mM gluconate, 0.2% casamino acids, and 2.5 mM *p-*coumaric acid (potassium salt). After 7h, cerulenin was added at final concentration 50 μM. Error bars represent the mean ± s.d of three biological replicates.

**Table S1:** List of strains and plasmids

| Strains and Plasmids | Relevant characteristics | Reference or source |
| --- | --- | --- |
| *E. coli* XL1-Blue | Wild-type *E. coli* XL1-Blue | Stratagene, USA |
| *A. baylyi* ADP1 |  |  |
| ADP1 | Wild-type *A. baylyi* ADP1 | DSM 24193, DSMZ |
| ASA800 | ADP1 Δ*hcaA,* deletion of a 4-hydroxycinnamoyl CoA hydratase/lyase | This study |
| ASA801 | ADP1 Δ*hcaA –* pNAR1 | This study |
| ASA802 | ADP1 Δ*hcaA –* pNAR2 | This study |
| ASA803 | ADP1 Δ*hcaA –* pNAR3 | This study |
| ASA804 | ADP1 Δ*hcaA –* pNAR4 | This study |
| ASA805 | ADP1 Δ*hcaA –* pNAR5 | This study |
| ASA806 | ADP1 Δ*hcaA –* pNAR6 | This study |
| ASA807 | ADP1 Δ*hcaA –* pBAV1C-chn | This study |
| ASA808 | ADP1 *–* pNAR3 | This study |
| ASA809 | ADP1 *–* pNAR6 | This study |
| ASA810 | Δ*hcaA* Δ*mdcABCDEGH* Δ*poxB* Δ*metY* Δ*acr1*::*Rt*matB-Kan^R^ - pNAR3, | This study |
|  | Δ*hcaA* strain with deletion of malonate degradation pathway (*mdcABCDEGH*), *poxB*, *metY*, *acr1*, integration of *matB*, and expression of pNAR3 |  |
| ASA811 | Δ*mdcABCDEGH* Δ*poxB* Δ*metY* Δ*acr1*::*Rt*matB-Kan^R^-pNAR3, | This study |
|  | Deletion of malonate degradation pathway (*mdcABCDEGH*), *poxB*, *metY*, *acr1*, integration of *matB*, and expression of pNAR3 |  |
| Plasmids |  |  |
| pBAV1C-chn | High copy number, Cm^r^, cyclohexanone inducible promoter (*P_chnB_)*, rrnB T1 terminator | (Luo et al., 2019) |
| pIX/pIM1463 | Cm^r^, Kan^R^, T5 promoter, integrative plasmid, knock-out pyruvate dehydrogenase (*poxB*), homocysteine synthase (*metY*), fatty acyl-CoA reductase (*acr1*) (ACIAD3383-3381) | (Lehtinen et al., 2018) |
| pNAR1 | pBAV1C-chn carrying codon-optimized *P. hybrida* CHS and codon-optimized *P. lobata* CHI | This study |
| pNAR2 | pBAV1C-chn carrying codon-optimized *H. serrata* PKS1 and codon-optimized *P. lobata* CHI | This study |
| pNAR3 | pBAV1C-chn carrying codon-optimized *H. androsaemum* CHS and codon-optimized *P. lobata* CHI | This study |
| pNAR4 | pBAV1C-chn carrying codon-optimized *P. hybrida* CHS and codon-optimized *M. sativa* CHI | This study |
| pNAR5 | pBAV1C-chn carrying codon-optimized *H. serrata* PKS1 and codon-optimized *M. sativa* CHI | This study |
| pNAR6 | pBAV1C-chn carrying codon-optimized *H. androsaemum* CHS and codon-optimized *M. sativa* CHI | This study |
| pIX-matB | pIX integration cassette carrying codon-optimized *R. trifolii* matB-Kan^R^, Cm^R^ | This study |

**Table S2:** Primers used in this study

| Primers | Sequences (5' to 3') | Purpose |
| --- | --- | --- |
| R1_hcaA_FW | gacatgatcccgaacagcataatactc | hcaA deletion |
| R1_hcaA_RV | TTTTTATGATTTGAATTGGAGGCTGGGttgttttctccttgaatgacaAATTGTGC |  |
| R2_hcaA_FW | CGATGAGTTTTTCTAAGCATGCGGAGCTGGgttCGCAACATCAATGCTCaaG |  |
| R2_hcaA_RV | ATTGCAAGTGCTCGGGTTG |  |
| Ver_hcaA_FW | CAGCTTCTGATGCGACCGTAATC | hcaA verification |
| Ver_hcaA_RV | CATGTGTAATGACATTAACCACACCTTCAC |  |
| R1_hcaA_rsc_RV | acctagggcggccgccaattgattgttttctccttgaatgacaAATTGTGC | hcaA rescue cassette |
| R2_hcaA_rsc_FW | tcaattggcggccgccctaggtgttcgcaacatcaatgctcaag |  |
| R1_mdcA-H_FW | CAGCAATCTTCCCAAACTCAT | mdcABCDEGH deletion |
| R1_mdcA-H_RV | TTTTTATGATTTGAATTGGAGGCTGGGgtacgtttccttgtgaatattttg |  |
| R2_mdcA-H_FW | CGATGAGTTTTTCTAAGCATGCGGAGCTGGagcataatcagaaaataatgaaactc |  |
| R2_mdcA-H_RV | tgctgtaattaaacctaaatgagt |  |
| Ver_mdcA-H_FW | GAATAATCACCCAATTGCAAAGC | mdcABCDEGH verification |
| Ver_mdcA-H_RV | gttccacctacccaagcc |  |
| R1_mdcA-H_rsc_RV | acctagggcggccgccaattgaGTAcgtttccttgtgaatattttg | mdcABCDEGH rescue cassette |
| R2_mdcA-H_rsc_FW | tcaattggcggccgccctaggtAGCataatcagaaaataatgaaactc |  |
| 3381_v1 | ttggctaacttgtcaaagtc | matB verification in the genome |
| 3381_v2 | tagagtgtagaaacagatgc |  |

underline: overlap with tdk-kan cassette

**Table S3:** Summary of naringenin production in the fed-batch process.

| **Bioreactor parameter** | **Strain** | **Titer (mg/L)*** | **Yield (g_naringenin_/g*_p-_*_coumaric-acid_)*** | **Productivity (mg/L/h)*** |
| --- | --- | --- | --- | --- |
| Feeding with 50 mM gluconate, 0.2% casamino acids, 3.2 mM *p-*coumaric-acid for 48h | | | | |
| pH 7.5 | ASA803 | 36.79 | 0.29 | 0.37 |
|  | ASA806 | 34.24 | 0.31 | 0.48 |
| No pH control | ASA803 | 47.59 | 0.45 | 0.66 |
|  | ASA806 | 60.03 | 0.54 | 0.83 |
|  |  |  |  |  |
| Feeding with 100 mM gluconate, 0.2% casamino acids, 3.2 mM *p-*coumaric-acid for 48h | | | | |
| No pH control | ASA806 | 66.37 ** | 0.32 ** | 0.65 ** |

*) after 72 h cultivation

**) after 102 h cultivation

**Table S4:** Nucleic acid sequences of synthetic genes used in this study. The sequences are codon optimized for expression in *A. baylyi* ADP1.

| Genes/genetic components | Sequences (5' to 3') |
| --- | --- |
| RBS (BBa_B0034) | AAAGAGGAGAAA |
| ChnB promoter | GCAACTAAAAGAGATTGTTTGGATCAGTTACCCAAAATCGTTGAAAAGATTTTAACTCTTCGATTTTTATTTTTTAGGTAATCCTAGCCCTCTCGGGGGCTAGGATTAAAAATTTTAAGTTATTCCAACACGAATGACAAATTGTTCAATGCAAAATAAAAACATACAATATATAAATATATTTTTTAAATAAAACATAAGATTACAATAAAATAAGAATTTTTATTTGGAGTTTGTTTTTTTTCTACAATGATCATTATGTACAATTTTTAGGTTCACCCCATCCAAGCCTTGTGATTGCATTCCTGCGATTCTTTATTCAATGAATAAGCAATGCTATTAATCAGCAATGAATAACCAGCAGTGCAGATTTTGAATAAATTCACATGTCGTAAT |
| Km promoter | ACGTCTTGTGTCTCAAAATCTCTGATGTTACATTGCACAAGATAAAAATATATCATCATGAACAATAAAACTGTCTGCTTACATAAACAGTAATACAAGGGGTGTT |
| T5 promoter | CTTTGCTCAAAGAATCATAAAAAATTTATTTGCTTTCAGGAAAATTTTTCTGTATAATAGATTCAAATTGTGAGCGGATAACAATTTGAATTTC |
| rrnB T1 terminator | ATAAAACGAAAGGCTCAGTCGAAAGACTGGGCCTTTCGTTTTAT |
| ChnR | ATGAGCACAGACAAAGCAAATACGCTGATCAAACCCGAAGATGTCGTGTTATGGATTCCGGGTAATGTCACAATTGACAGCATGAATGCCGGTTGGGAAAACATTGCAATCAGAGGGTACGAATATACCAACCTCGATGTGCATATTCCTGCCATGCGTGACTACATGATCGTCAACTATAAAAAAAGTGCGGCGGAAATGCGTAGAAAAGGCGATGCCTCTTGGGATACCCAAGTGGTTAAGCCGGGTTATGTCTCCTTGTTGACCTGTGGTGAAGATTCCCGCTGGGCGTGGAATGACCATATTGCCGTCACCCATGTCTACATTTCGCATGACTCCATCACCTCAATGGCGAATAAGGTGTTTGATTATGATATCGCTTCGATCCGAATCAGAGACGAAGTCGGTGTGGAAGATCATGTTTTACCTGCTCTGACTTCACTTTTAGAACTAGAATTAAAGCAAGGTGGTTTAGGTGGAAACCTGTATTTAGAGAGCATTAAAAACCAGATCGCCCTGCATTTACTCCGTCAGTATGCCAAATTAGATTTTAAGGAAGGACAGTGCCGTTCTGGTTTTACTCCCCTACAACGCAGACTGTTATTAGAATTTATCAATGAAAACATGAGCATTAAAATTACCCTCGAAGATTTAGCGGGATTAGTCAAGATGAGCGTGCCTCATTTAATGAGAAAATTTAAAGTCGATTTTGGTAATTCCCCTGCTGCCTACATCATGAATCTCAGGGTGCAATTTGCTAAACGTTTGCTCACTTCAAAAAAAGAAATTCCACTGAAAGTGATTGCCAGTGAAGCCGGTTTTTGCGATCAGAGCCATATGACCCGAGTATTTCAAAAATTTTTTGGGAAAACACCCATCGAAATCAGACAGGAACACACCAATCTCGTGTCTGAAAATTCAGTCTCCTCTATTGTTTTTTGA |
| PhCHS | ATGGTGACCGTAGAAGAATATCGTAAAGCACAACGTGCAGAAGGTCCAGCAACTGTGATGGCTATTGGTACAGCAACTCCAACAAATTGTGTAGATCAAAGTACATATCCTGATTATTATTTTCGTATTACTAATTCAGAACATAAAACAGATCTGAAAGAAAAATTTAAACGTATGTGTGAAAAATCTATGATTAAAAAACGTTATATGCATTTAACCGAAGAAATTTTAAAAGAAAATCCAAGTATGTGTGAATATATGGCACCTTCATTAGATGCACGTCAAGATATTGTTGTGGTAGAAGTGCCAAAATTGGGTAAAGAAGCAGCACAAAAAGCAATTAAAGAATGGGGTCAACCTAAATCAAAAATTACTCATCTGGTGTTTTGTACAACCTCTGGTGTGGATATGCCAGGTTGTGATTATCAATTAACAAAATTATTAGGTCTGCGTCCTAGTGTAAAACGTCTGATGATGTATCAACAAGGTTGTTTTGCAGGTGGTACAGTTTTACGTCTGGCAAAAGATCTGGCAGAAAATAATAAAGGTGCACGTGTTTTAGTTGTGTGTTCTGAAATTACCGCAGTGACTTTTCGTGGTCCAAATGATACTCATTTAGATAGTTTAGTTGGTCAAGCATTATTTGGTGATGGTGCAGGTGCAATTATTATTGGTAGTGATCCAATTCCTGGTGTAGAACGTCCATTATTTGAATTAGTTAGTGCAGCACAAACATTATTACCTGATAGTCATGGTGCTATTGATGGTCATTTACGTGAAGTAGGTCTGACCTTTCATTTATTAAAAGATGTTCCAGGTCTGATTTCAAAAAATATTGAAAAATCTTTAGAAGAAGCATTTCGTCCTCTGTCTATTAGTGATTGGAATAGTTTATTTTGGATTGCACATCCAGGTGGTCCTGCAATTTTAGATCAAGTGGAAATTAAATTGGGTCTGAAACCAGAAAAATTGAAAGCAACTCGTAATGTTCTGTCAAATTATGGTAATATGAGTTCAGCATGTGTGCTGTTTATTTTAGATGAAATGCGTAAAGCATCTGCAAAAGAAGGTTTAGGTACTACAGGTGAAGGTTTAGAATGGGGTGTATTATTTGGTTTTGGTCCTGGTTTAACAGTGGAAACCGTAGTTTTACATTCAGTAGCAACATAA |
| HsPKS1 | ATGACAATTAAAGGTTCTGGTAGTGCAGCATTTGAAGGTACACGTTTATGTCCACGTGTTATTAAACCAGATGGTCCTGCAACCATTTTAGCTATTGGTACAAGTAATCCAACAAATATTTTTGAACAAAGTACTTATCCTGACTTTTTCTTTGATGTGACAAATTGTAATGATAAAACTGAATTAAAGAAAAAATTTCAACGTATTTGTGATAAAAGTGGTATTAAAAAACGTCATTTTCATTTAACAGATGAAATTTTACGTAAAAATCCAAGTATTTGTAAATTTAAAGAAGCATCATTAGATCCACGTCAAGATATTGCAGTATTAGAAGTTCCTAAATTAGCAAAAGAAGCAGCAATTTCAGCAATTAAACAATGGGGTCAACCTAAATCTAAAATTACCCATCTGGTATTTGCAACAACCTCTGGTGTTGATATGCCAGGTGCAGATTTTCAATTAGCAAAATTATTAGGTCTGCGTCCTACTGTGAAACGTGTAATGTTATATCAACAAGGTTGTTATGCAGGTGCAACAGTTTTACGTGTGGCAAAAGATCTGGCAGAAAATAATAAAGGTGCACGTGTTTTAGTGGCATGTAGTGAAGTAACCGCAGTTACTTTTCGTGCACCAAGTGAAACTCATTTAGATGGTTTAGTTGGTTCAGCATTATTTGGTGATGGTGCAGCAGCATTAATTGTTGGTAGTGATCCAGTGCCTCAAGAAGAAAAACCACTGTTTGAAATTCATTGGGCAGGTGAAGCAGTATTACCTGATTCAGATGGTGCAATTAATGGTCATTTACGTGAAGCAGGTTTAATTTTTCATTTATTAAAAGATGTTCCTGGTCTGATTTCAAAAAATATTGATAAAGTGCTGGCAGAACCATTAGAATATGTACATTTTCCTTCTTATAATGATATGTTTTGGGCAGTGCATCCAGGTGGTCCTGCAATTTTAGATCAAATTGAAGCAAAATTGGGTCTGTCAACCGATAAAATGCAAGCATCACGTGATGTACTGGCATCTTATGGTAATATGAGTTCAGCATCTGTGCTGTTTGTATTAGATCAAATTCGTAAAAATAGTGAAGAATTACATTTACCAACTACAGGTGAAGGTTTTGAATGGGGTTTTGTTATTGGTTTTGGTCCTGGTTTAACAGTGGAAACCTTATTATTACGTTCTATTAATATCTAA |
| HaCHS | ATGGTGACTGTAGAAGAAGTACGTAAAGCACAACGTGCAGAAGGTCCAGCAACCGTTATGGCTATTGGTACAGCAGTTCCACCAAATTGTGTGGATCAAGCAACATATCCTGATTATTATTTTCGTATTACCAATAGTGAACATAAAGCAGAATTAAAAGAAAAATTTCAACGTATGTGTGATAAATCACAAATTAAAAAACGTTATATGTATCTGAATGAAGAAGTTTTAAAAGAAAATCCAAATATGTGTGCATATATGGCACCTAGTTTAGATGCACGTCAAGATATTGTTGTGGTAGAAGTGCCAAAATTGGGTAAAGAAGCAGCAGTAAAAGCAATTAAAGAATGGGGTCAACCTAAATCTAAAATTACCCATCTGGTATTTTGTACAACCAGTGGTGTTGATATGCCAGGTGCAGATTATCAATTAACTAAATTATTAGGTCTGCGTCCTTCAGTTAAACGTCTGATGATGTATCAACAAGGTTGTTTTGCAGGTGGTACAGTGTTACGTCTGGCAAAAGATCTGGCAGAAAATAATAAAGGTGCACGTGTTTTAGTTGTGTGTTCTGAAATTACAGCAGTGACCTTTCGTGGTCCAACCGATACTCATTTAGATAGTTTAGTAGGTCAAGCATTATTTGGTGATGGTGCAGCAGCAATTATTATTGGTAGTGATCCAATTCCTGAAGTGGAAAAACCACTGTTTGAATTAGTAAGTGCAGCACAAACTATTTTACCTGATTCAGAAGGTGCTATTGATGGTCATTTACGTGAAGTTGGTCTGACATTTCATTTATTAAAAGATGTTCCAGGTCTGATTTCAAAAAATGTGGAAAAATCTTTAACAGAAGCATTTAAACCTTTAGGTATTTCTGATTGGAATAGTTTATTTTGGATTGCACATCCAGGTGGTCCTGCAATTTTAGATCAAGTGGAAGCAAAATTATCATTAAAACCAGAAAAATTGCGTGCAACCCGTCATGTGCTGTCTGAATATGGTAATATGAGTTCAGCATGTGTACTGTTTATTTTAGATGAAATGCGTCGTAAATCAAAAGAAGATGGTCTGAAAACTACAGGTGAAGGTATTGAATGGGGTGTTTTATTTGGTTTTGGTCCTGGTCTGACTGTAGAAACAGTAGTTCTGCATTCTGTTGCAATCAATTAA |
| PlCHI | ATGGCAGCAGCAGCAGCAGTTGCAACTATTTCAGCAGTACAAGTTGAATTTTTAGAATTTCCAGCAGTTGTGACATCACCTGCATCTGGTCGTACCTATTTTCTTGGTGGTGCAGGTGAACGTGGTTTAACTATTGAAGGCAAATTTATTAAATTTACAGGTATTGGTGTATATTTAGAAGATAAAGCAGTTAGTTCATTAGCAGCAAAATGGAAAGGTAAACCATCTGAAGAATTAGTAGAAACATTAGATTTTTATCGTGATATTATTTCAGGTCCATTTGAAAAATTGATTCGTGGTTCTAAAATTCTGCCTCTGTCTGGTGTAGAATATAGTAAGAAAGTTATGGAAAATTGTGTGGCACACATGAAAAGTGTTGGTACTTATGGTGATGCAGAAGCAGCAGCTATTGAAAAATTTGCAGAAGCATTTAAAAATGTTAATTTTCAACCAGGTGCAACCGTGTTTTATCGTCAAAGTCCTGATGGTGTATTAGGTTTATCTTTTAGTGAAGATGTTACTATTCCTGATAATGAAGCAGCAGTGATTGAAAATAAAGCAGTGAGTGCAGCAGTATTAGAAACAATGATTGGTGAACATGCAGTGTCACCAGATTTAAAACGTAGTTTAGCATCACGTTTACCAGCAGTTTTAAGTCATGGTATTATTGTATAA |
| MsCHI | ATGGCAGCATCAATTACAGCAATTACCGTGGAAAATTTAGAATATCCAGCAGTTGTGACTTCTCCTGTAACAGGTAAAAGTTATTTTCTTGGTGGTGCAGGTGAACGTGGTTTAACCATTGAAGGCAATTTTATTAAATTTACTGCTATTGGTGTTTATTTAGAAGATATTGCAGTGGCATCTTTAGCAGCAAAATGGAAAGGTAAAAGTTCAGAAGAATTATTAGAAACTTTAGATTTTTATCGTGATATTATTAGTGGTCCATTTGAAAAATTGATTCGTGGTTCAAAAATTCGTGAATTAAGTGGTCCTGAATATTCACGTAAAGTGATGGAAAATTGTGTAGCACATCTGAAAAGTGTTGGTACTTATGGTGATGCAGAAGCAGAAGCAATGCAAAAATTTGCAGAAGCATTTAAACCAGTAAATTTTCCACCTGGTGCAAGTGTGTTTTATCGTCAATCTCCTGATGGTATTTTAGGTTTATCATTTTCTCCAGATACATCTATTCCTGAAAAAGAAGCAGCACTGATTGAAAATAAAGCAGTATCTAGTGCAGTTTTAGAAACCATGATTGGTGAACATGCAGTGAGTCCAGATTTAAAACGTTGTTTAGCAGCACGTTTACCTGCATTATTAAATGAAGGTGCATTTAAAATTGGTAATTAA |
| RtMatB | TACCATATGGTTAGCAACCACCTGTTCGATGCGATGCGTGCTGCGGCGCCGGGTAACGCGCCGTTTATCCGTATTGATAACACCCGTACCTGGACCTATGACGATGCGTTCGCGCTGAGCGGTCGTATTGCGAGCGCGATGGACGCGCTGGGTATTCGTCCGGGCGATCGTGTGGCGGTTCAGGTGGAAAAGAGCGCGGAGGCGCTGATTCTGTACCTGGCGTGCCTGCGTAGCGGTGCGGTTTACCTGCCGCTGAACACCGCGTATACCCTGGCGGAACTGGACTACTTTATTGGTGATGCGGAACCGCGTCTGGTGGTTGTGGCGAGCAGCGCGCGTGCGGGCGTGGAAACCATCGCGAAGCCGCGTGGTGCGATTGTTGAGACCCTGGACGCGGCGGGTAGCGGCAGCCTGCTGGACCTGGCGCGTGATGAACCGGCGGACTTCGTGGATGCGAGCCGTAGCGCGGACGATCTGGCGGCGATCCTGTATACCAGCGGCACCACCGGCCGTAGCAAAGGTGCGATGCTGACCCACGGCAACCTGCTGAGCAACGCGCTGACCCTGCGTGACTTTTGGCGTGTTACCGCGGGTGATCGTCTGATCCACGCGCTGCCGATTTTCCACACCCACGGCCTGTTTGTGGCGACCAACGTTACCCTGCTGGCGGGTGCGAGCATGTTCCTGCTGAGCAAGTTTGACCCGGAGGAAATTCTGAGCCTGATGCCGCAGGCGACCATGCTGATGGGCGTGCCGACCTTCTACGTTCGTCTGCTGCAAAGCCCGCGTCTGGATAAACAAGCGGTGGCGAACATCCGTCTGTTCATTAGCGGTAGCGCGCCGCTGCTGGCGGAAACCCACACCGAGTTTCAAGCGCGTACCGGCCACGCGATCCTGGAACGTTATGGTATGACCGAGACCAACATGAACACCAGCAACCCGTACGAAGGCAAACGTATTGCGGGCACCGTTGGTTTCCCGCTGCCGGATGTTACCGTGCGTGTTACCGATCCGGCGACCGGTCTGGCGCTGCCGCCGGAACAAACCGGTATGATCGAGATTAAGGGCCCGAACGTTTTCAAAGGTTATTGGCGTATGCCGGAAAAGACCGCGGCGGAGTTTACCGCGGACGGCTTCTTTATCAGCGGTGATCTGGGCAAAATTGACCGTGATGGTTACGTGCACATCGTTGGTCGTGGCAAGGACCTGGTGATCAGCGGTGGCTACAACATTTATCCGAAAGAGGTTGAAGGCGAGATCGACCAGATTGAAGGTGTTGTTGAGAGCGCGGTGATTGGCGTTCCGCACCCGGATTTCGGTGAAGGCGTGACCGCGGTTGTGGTTCGTAAGCCGGGTGCGGCGCTGGACGAGAAAGCGATTGTTAGCGCGCTGCAAGATCGTCTGGCGCGTTACAAGCAACCGAAACGTATCATTTTTGCGGAGGACCTGCCGCGTAACACGATGGGTAAAGTTCAGAAGAACATCCTGCGTCAGCAATACGCGGATCTGTATACCCGTACCTAACTCGAG |

# References

Lehtinen, T., Efimova, E., Santala, S., Santala, V., 2018. Improved fatty aldehyde and wax ester production by overexpression of fatty acyl-CoA reductases. Microb Cell Fact 17, 19. https://doi.org/10.1186/s12934-018-0869-z

Luo, J., Lehtinen, T., Efimova, E., Santala, V., Santala, S., 2019. Synthetic metabolic pathway for the production of 1-alkenes from lignin-derived molecules. Microb Cell Fact 18, 48. https://doi.org/10.1186/s12934-019-1097-x
